# Adaptive Estimation Algorithm for Correcting Low-Cost MEMS-SINS Errors of Unmanned Vehicles under the Conditions of Abnormal Measurements

**DOI:** 10.3390/s21020623

**Published:** 2021-01-17

**Authors:** Lifei Zhang, Proletarsky Andrey Viktorovich, Maria Sergeevna Selezneva, Konstantin Avenirovich Neusypin

**Affiliations:** Department of Informatics and Control Systems, Bauman Moscow State Technical University, 101000 Moscow, Russia; erinzhangs@gmail.com (L.Z.); pav_mipk@mail.ru (P.A.V.); ms.selezneva@bmstu.ru (M.S.S.)

**Keywords:** strap-down inertial navigation system, unmanned vehicle, estimation algorithm, criterion for detecting abnormal measurements, accuracy analysis

## Abstract

In this paper, a low-cost small-sized strap-down inertial navigation system (SINS)—Gyrolab GL-VG 109—is studied. When the system is installed on an unmanned vehicle and works in autonomous mode, it is difficult to determine the navigation parameters of the unmanned vehicle. Correcting the SINS information from the Global Navigation Satellite System (GNSS) can significantly increase the determination accuracy of the navigation parameters. However, this is only available when the GNSS signals are stable. A new adaptive estimation algorithm that can automatically detect, evaluate, and process the abnormal measurements is proposed in the present work. The determination of the navigation parameters can reach the third accuracy class using the proposed method. The effectiveness of the algorithm is verified by the mathematical simulation and the experimental tests (with a real SINS GL-VG 109), which are conducted in urban environments with a GNSS signal containing 15% and 40% abnormal measurements. The results show that the proposed method can significantly reduce the impact of abnormal measurements and improve the estimation accuracy.

## 1. Introduction

In recent years, the emerging navigation and positioning technologies have greatly promoted the development and application of unmanned vehicles [1]. Generally, the inertial navigation system (INS) is usually selected as the basic navigation system because of its full autonomy, good concealment, and no susceptibility to interference [2]. However, INSs have measurement errors caused by inaccurate initial information, gyroscope drifts, accelerometer bias, etc. These errors will accumulate over time when determining the navigation information [3].

According to the navigation accuracy, INSs can be divided into several classes: high accuracy—the gyro drift is 0.016 grad/h; middle accuracy—0.05 grad/h; low accuracy—0.4 grad/h, which correspond to the first, second, and third accuracy classes [4]. There are also small-sized low-cost strap-down inertial navigation systems (SINSs) based on microelectromechanical systems (MEMSs), the drift of which is about 15–100 grad/h. Although the accuracy of the MEMS-SINS is relatively low and varies over a wide range as a result of the instability of MEMS elements, it is still widely used by the military and civilians because of its extremely low price [5]. This kind of INS is usually installed on small unmanned aerial vehicles (UAVs) and unmanned vehicles (UVs) with a limited life cycle [6,7,8], which are often single use and play an important role in the case of chemical and radioactive contamination, mine clearance, fire fighting, and natural or technological disasters [9]. The aim of the present work is to study the characteristics of the MEMS-SINS and propose a method to improve the positioning accuracy.

In order to improve the accuracy of the MEMS-SINS, two methods are usually used: design a more accurate SINS or use the algorithmic method to correct the SINS errors [10]. The first method involves the availability of new technology, which will take a long time and require serious financial costs, while the second method can achieve the desired accuracy in a short period of time with minimal financial costs. Additionally, using the algorithmic method does not limit the implementation of new design solutions. Therefore, the modern SINSs are distinguished by a large amount of algorithmic support.

The algorithmic correction of SINS accumulation errors is usually carried out by using the external measurement information such as the Global Navigation Satellite System (GNSS) [11]. The GNSS has a sufficiently high long-term accuracy and is distinguished by its high precision, high efficiency, signal availability, and integrity [12]. Integrating the SINS with the GNSS can significantly increase the navigation accuracy [13]. However, the GNSS signal is sensitive to interference. In urban environments, the GNSS positioning accuracy may be degraded because of the following factors: satellite signal blockage due to buildings, bridges, and trees; signals reflected off buildings or walls (“multipath”) [14,15]. In this case, the GNSS signals may contain 10–30% abnormal measurements in the form of a single outlier or short-term or long-term outliers [15]. The abnormal measurements should be processed properly, otherwise they will greatly reduce the accuracy of the SINS/GNSS integrated navigation system or even lead to divergence.

There are several methods to process the GNSS signal under the conditions of abnormal measurements: moving average algorithm [16,17], median filter [18,19,20], Tukey 53X procedure [21,22,23], the adaptive Kalman filter that can identify and adjust noise statistics online [24,25,26], etc. The average positioning accuracy (root mean squared (RMS)) obtained by different methods in a large number of outdoor experiments is shown in Table 1. The moving average algorithm is a linear method, which is effective for suppressing small amplitude high frequency noise, but not applicable for occasional impulsive noise. It can significantly smooth the GNSS measurements, but cannot completely eliminate the effect of outliers [16]. The nonlinear algorithms, median filter, and Tukey 53X procedure could partially remove the outliers, but when continuous outliers occur, the performance of the algorithms degrades [18,27]. In addition, these methods are not suitable for high dynamic conditions due to the aging effect of measurements [28]. As show in Table 1, the adaptive Kalman filter has the highest accuracy. This is because the adaptive filters can predict state variables according to the model and can automatically adapt to the model errors, inaccurate prior statistical information, and changing noise. The most common adaptive filters modify the covariance matrix of the input and measurement noises through the innovation sequence and then adjust the utilization ratio of the model prediction value and the measurement information [24,29].

These kinds of adaptive Kalman filters have a good estimation performance under dynamic conditions. However, their suppression effect on abnormal measurements is limited. In order to quickly and effectively detect the abnormal measurement values, a criterion is introduced in Section 6. When abnormal measurements are detected, we propose a method to restrain the value of measurement information. The new adaptive Kalman filter can work in the absence of accurate prior statistics of noise and under the conditions of abnormal measurements. The conceptual idea of adaptation is to use the information contained in the innovation sequence [24]. The adaptive determination of the covariance matrices of noise and the criterion for detecting abnormal measurements can be developed from Jazwinski’s idea of using the innovation sequence. These two methods lead to the creation of the proposed adaptive estimation algorithm.

The original feature of this algorithm is the relay selection of the formula for estimating x. If an abnormal measurement is detected, the estimate is calculated using the maximum theoretically predicted value of the innovation sequence (Formulas (9) and (10)). If the criterion does not detect an abnormal measurement, then the estimate is calculated as in the conventional adaptive estimation algorithm (Formulas (5)–(7)). The simulation and experimental results show that with the help of the GNSS and the proposed estimation algorithms, it is possible to obtain navigation information of a higher accuracy class with a low-cost low-accuracy SINS.

The paper is organized as follows. In Section 2, the low-cost strap-down inertial navigation system based on MEMS sensors is introduced, and in Section 3 and Section 4, the characteristics of the SINS and GNSS error sources are analyzed, which is important for the design of estimation algorithms. In Section 5, the correction scheme for SINS errors using the estimation algorithm is introduced. In Section 6, a new adaptive estimation algorithm that can work under the conditions of abnormal measurements is proposed. The simulation results and experimental tests are shown in Section 7 and Section 8.

## 2. Strap-Down Inertial Navigation Systems

Compared to the traditional stable platform inertial navigation systems, the SINSs have the following advantages: greater reliability, smaller size, convenience in application, and lower cost [11,30]. The strap-down inertial navigation system is a frameless system without gyro-stabilized platforms and consists of six or three accelerometers, two three-degree gyroscopes, and a microcomputer. The inertial sensors of the SINS are strapped down rigidly to the moving vehicle. The mechanical stabilization that was provided within stable-platform systems is replaced by a computational model to achieve the same output navigation states [31]. Obtaining the navigation information relative to the selected coordinate system, the SINSs model a mathematical pendulum with a Schuler period using the information about the linear accelerations and angular velocities of the carrier.

Since the sensitive elements of SINS are rigidly fixed directly to the carrier, the accuracy requirements for accelerometers and gyroscopes are relatively high considering that the UVs may work in extremely harsh conditions. In order to prevent the error from increasing rapidly, high-precision accelerometers and gyroscopes should be used when building SINSs, and a computer with a high computing speed is required to carry out a significant amount of the calculations. Currently, with the development of new gyroscope technologies and the widespread use of high-performance computers, it is possible to design reliable and economical SINSs with the required accuracy.

Among the various types of gyroscopes (such as laser, fiber optic, microelectromechanical, piezoelectric, vibrational, etc.), the microelectromechanical gyroscopes are widely used because of their low price. The advent of MEMS technologies has significantly reduced the cost of the SINS [4,32,33], but at the same time has also sacrificed accuracy.

**Figure 1:**
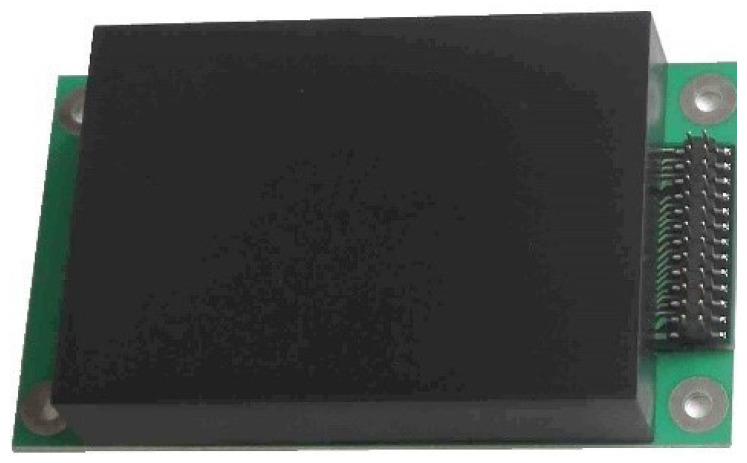
MEMS-strap-down inertial navigation system (SINS) GL VG 109.

In this work, a low-cost low-accuracy SINS based on MEMS sensors—GL-VG 109—is studied. The equipment is shown in Figure 1, and its parameters are presented in Table 2.

## 3. SINS Errors in Autonomous Mode

In practical applications, SINSs could be used in autonomous mode or combined with other external sensors to form an integrated navigation system. The main advantage of autonomous SINSs is their invariance to horizontal acceleration. However, the errors of autonomous SINSs due to the drift of gyroscopes, the zero offset, and the drift of accelerometers, as well as other disturbing factors can reach significant values.

SINS errors can be divided into two types: methodological and instrumental. Methodological SINS errors are caused by the methods of measurement. They usually include errors caused by the inaccurate values of the structure, the parameters of the Earth’s gravitational field, and the quantitative characteristics of its shape. Errors due to the simplification of algorithms should also be included here. Usually, most methodological errors can be successfully compensated.

Instrumental errors are caused by inertial sensor errors and computing devices, including, for example, the random drift of gyroscopes, the instability of the sensor scale factors of gyroscopes and accelerometers, and the errors of information transfer. The causes of a number of other errors are due to structural and technological factors: errors in the performance of landing bases for inertial sensors, as well as the instability of the relative positions of these bases, errors in the initial calibration that consist of inaccuracy of external information, and errors of devices in inputting this information to the SINS.

Considering the reaction of an autonomous SINS to certain disturbing factors, we can draw conclusions about the characteristics of SINS errors. The dominant influence on the total SINS error in determining the distance traveled is exerted by the gyroscope drift velocities. The systematic drift velocity of the gyroscopes causes the increasing-over-time component of SINS errors, as well as the oscillatory component of the Schuler period. The increasing-over-time drift velocity causes the appearance of the SINS error, which can also be represented in the form of two components. The first component changes in proportion to the square of the SINS operation time, and the second component oscillates with the Schuler period.

The SINS error model for one horizontal channel is as follows [34,35,36].

(1) $$x_{k+1}=\Phi_{k+1,k}x_{k}+W_{k}$$

where $x_{k}$ is the state vector, $\Phi_{k+1,k}$ is the state-transition matrix, $W_{k}$ is the input noise vector, and $x_{k}=\left[\begin{array}{c} \delta V_{k}\\ \varphi_{k}\\ \varepsilon_{k} \end{array}\right];\Phi =\left[\begin{array}{ccc} 1 & -gT & 0\\ \frac{T}{R} & 1 & T\\ 0 & 0 & 1-\beta T \end{array}\right];W_{k}=\left[\begin{array}{c} B\\ 0\\ w_{k} \end{array}\right]$.

Here, $\delta V_{k}$ is the INS errors in determining the velocity, $\varphi_{k}$ is the deviation angles of the computing coordinate system relative to the accompanying one, $\varepsilon_{k}$ is the gyro drift velocity, *g* is the acceleration of gravity, *B* is the zero offset of the accelerometer, $B=10^{-2}$, *R* is the radius of the Earth, *T* is the sampling period, β is the average frequency of random changes in the gyro drift, and $w_{k-1}$ is the discrete analog of white Gaussian noise with zero meanand covariance matrix $E\left[W_{j}W_{k}^{T}\right]=Q_{k}\delta_{j,k}$, where $Q_{k}$ is a positive semi-definite matrix, $\delta_{j,k}$ is the Kronecker symbol.

It is assumed that only the first component of the state vector is measured, i.e.,

(2) $$z_{k}=H_{k}x_{k}+V_{k}$$

where $z_{k}$ is the measurement vector, $H_{k}$ is the observation matrix, and $H_{k}=\left[\begin{array}{ccc} 1 & 0 & 0 \end{array}\right]$; $V_{k}$ is the measurement noise, which is a discrete analog of white Gaussian noise with zero meanand covariance matrix $E\left[V_{j}V_{k}^{T}\right]=R_{k}\delta_{j,k}$, where $R_{k}$ is a positive semi-definite matrix. Measurement noise $V_{k}$ and input disturbances $W_{k-1}$ are uncorrelated. The initial value of the state vector is assumed to be a Gaussian random vector with zero mean and is independent of the input disturbances of the measurement noise: for any *k*, $E\left[x_{0}W_{k}^{T}\right]=0$, $E\left[x_{0}V_{k}^{T}\right]=0$.

The given equations of the SINS error model are used later in the estimation algorithm.

## 4. The Characteristic Analysis of SINS and GNSS Errors

No matter what kind of moving objects the SINS is installed on, the working principle remains the same: the vehicle body coordinates are determined by integrating the motion equations of its center of mass in the inertial coordinate system. The acceleration of the center of mass is measured by accelerometers, the orientation of the sensitivity axes of which is carried out using gyroscopes. At the same time, the diversity of objects, the difference in their trajectories and parameters, and the time of motion determine the essential features of the SINS both from the perspectives of theory and technical implementation.

One of the main characteristics of the SINS is the operating time, i.e., the time for continuously solving navigation problems. In a long-term operation of the SINS, it becomes necessary to calibrate and subsequently consider the parameters of the gyro drift, transmission coefficients, and the zero offset of accelerometers. Usually, this is done directly on a moving object while the SINS is running.

The main disadvantage of the SINS is that its errors accumulate over time. Therefore, the external sensors are usually used to correct SINS errors and improve the accuracy of navigation. The correction of the SINS is often carried out by using the GNSS. However, GNSS signals also contain errors due to the poor noise immunity of the information channel. Therefore, the SINS and GNSS signals are usually subjected to joint processing in an on-board computer. By comparing the SINS and GNSS signals, it is possible to single out a mixture of errors of these systems. A signal proportional to these errors is used as an input to the estimation algorithm. We can calculate the SINS errors and filter the GNSS errors out by using the estimation algorithm. It is possible to isolate from a mixture of errors a signal proportional to the SINS errors on the basis that the GNSS and SINS signals have different physical nature.

The SINS signal has a low-frequency character, and the GNSS signal contains a pronounced high-frequency component. In the estimation algorithm, GNSS errors are taken as the measurement noise and are suppressed. From the output of the estimation algorithm, a signal proportional to the SINS error acts as the output signal of the SINS, where it is algebraically subtracted from the information signal proportional to the location and speed of the UV. In this way, the navigation information obtained with the help of SINS is corrected.

The advantages and disadvantages of the SINS and GNSS are summarized in Table 3:

Obviously, combining the SINS and GNSS information to build an integrated navigation system not only retains the advantages of each navigation system, but also significantly reduces the impact of their shortcomings.

## 5. The Correction of SINS Errors Using the Estimation Algorithm

The correction of the SINS from the external information sources using various algorithms can significantly reduce the SINS errors [37,38,39]. Let us see an example of using estimation algorithms to correct navigation information—the SINS integrated with the GNSS, which is used as the external sources of information.

**Figure 2:**
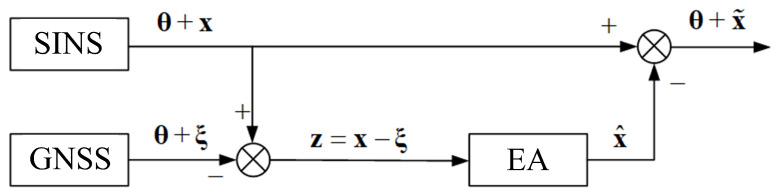
The correction scheme of SINS errors using the estimation algorithm (EA).

To completely compensate for errors in the output information, it is necessary to estimate the SINS errors first. The correction scheme of SINS errors with the estimation algorithm (EA) is presented in Figure 2. The estimates of all observable SINS errors could be obtained from the output of the estimation algorithms.

θ is the true information about the navigation parameters of the aircraft. *x* is the state vector consisting of the SINS errors. ξ is the GNSS measurement errors. *z* is the measurement vector, which is a mixture of SINS and GNSS errors. $\hat{x}$ is the state estimate of the SINS errors. $\tilde{x}$ is the estimation error.

As shown in Figure 2, the difference between the SINS’s and GNSS’s speed measurements is the input signal for the estimation algorithm. The state vector of the system consists of SINS errors such as errors in speed, angle, and gyro drift. GNSS errors are the measurement noise.

After processing the measurements, we obtain an estimate of the state vector at the output of the estimation algorithm, i.e., the estimation of all observable SINS errors. Further, the SINS error estimate is algebraically subtracted from the SINS output signal, which consists of reliable information about the speed and location of the UV and SINS errors. Thereby, the SINS errors in determining the navigation parameters are compensated in the output signal.

SINS error estimation can be used in the controller to compensate for errors in speed, angle, and gyro drift in the SINS structure to improve the quality of the transient response by reducing the amplitude of the error oscillations.

The application of the scheme (Figure 2) involves the use of non-diverging high-precision estimation algorithms that require a small amount of computer memory, which can easily be implemented on a digital computer.

The use of estimation algorithms to improve the accuracy of navigation information allows for correction in the output information signal without interfering with the SINS dynamics.

Currently, the Kalman filter (KF) and its various adaptive modifications are usually used as the estimation algorithms for correcting the SINS of the UV [40,41,42].

Consider a linear system describing the changes in SINS errors of the form of Equation (1).

Then, the optimal estimate of the state vector in the KF is determined as follows:

(3) $${\hat{x}}_{k+1}=\Phi_{k+1,k}{\hat{x}}_{k}+K_{k+1}v_{k+1},$$

where $K_{k+1}$ is the KF gain matrix, $v_{k+1}=z_{k+1}-H_{k+1,k}\Phi_{k+1,k}{\hat{x}}_{k}$ is the innovation sequence, and ${\hat{x}}_{k}$ is the estimate of the state vector.

Based on the estimation of the state vector and the matrix of the object, a forecast is made for the next step in calculating the estimate. At the same time, this forecast is corrected by using an innovation sequence. The innovation sequence is the sum of the forecast error and the measurement noise.

The gain matrix of the KF determines the weight, with which the innovation sequence is included in the state vector estimate. In the case of the ideal measurement, i.e., when there is no measurement noise, the gain matrix will be selected as the maximum value. The greater the measurement noise, the less weight of the innovation sequence is taken into account when forming the state vector estimate.

The KF has the following form [41,43]:

(4) $$\begin{array}{c} {\hat{x}}_{k+1}=\Phi_{k+1,k}{\hat{x}}_{k}+K_{k+1}v_{k+1}\\ P_{k+1|k}=\Phi_{k+1,k}P_{k}\Phi_{k+1,k}^{T}+Q_{k}\\ K_{k+1}=P_{k+1|k}H_{k+1}^{T}{\left[H_{k+1}P_{k+1|k}H_{k+1}^{T}+R_{k+1}\right]}^{-1}\\ P_{k+1}=\left(I-K_{k+1}H_{k+1}\right)P_{k+1|k} \end{array}$$

where $P_{k+1|k}$ is the a priori covariance matrix of estimation errors, $P_{k+1}$ is the a posteriori covariance matrix of estimation errors, and *I* is the identity matrix. Using the Kalman filter, not only the estimation of the entire system state vector is carried out, but also the influence of the measuring noise is suppressed.

Since in practice, the inaccurate a priori statistical information of input and measurement noise may lead to a degradation of filter performance or divergence, it is usually suggested to use the adaptive Kalman filter (AKF). By using the information contained in the innovation sequence, the covariance matrices of the input and measurement noise could be determined adaptively in AKF [24,28].

The adaptive covariance matrix of estimation errors in the adaptive estimation algorithm is determined by the following formula:

(5) $$P_{k+1|k}=\Phi_{k+1,k}P_{k}\Phi_{k+1,k}^{T}+K_{k}E\left[v_{k}v_{k}^{T}\right]K_{k}^{T}$$

The gain matrix for the adaptive estimation algorithm has the following form:

(6) $$K_{k+1}=\left\{\begin{array}{c} P_{k+1|k}H_{k+1}^{T}{\left[E\left(\hat{v_{k+1}v_{k+1}^{T}}\right)\right]}^{-1}\\ P_{k+1|k}H_{k+1}^{T}{\left[H_{k+1}P_{k+1|k}H_{k+1}^{T}+R_{k+1}\right]}^{-1} \end{array}\right.\mathrm{when}\left\{\begin{array}{c} diag\left[E\left(\hat{v_{k+1}v_{k+1}^{T}}\right)\right]>diag\left[H_{k+1}P_{k+1|k}H_{k+1}^{T}+R_{k+1}\right]\\ diag\left[E\left(\hat{v_{k+1}v_{k+1}^{T}}\right)\right]\leq diag\left[H_{k+1}P_{k+1|k}H_{k+1}^{T}+R_{k+1}\right] \end{array}\right.$$

The calculation of the mathematical expectation of the innovation sequence is calculated by the following formula:

(7) $$E\left(\hat{v_{k}v_{k}^{T}}\right)=\frac{1}{N}\sum_{i=k-N+1}^{k}\left(v_{i}v_{i}^{T}\right)$$

where *N* is the number of samples used. The length of the sliding window *N* is generally selected according to the actual conditions.

Through the above methods, the adaptive estimation algorithms can work well in the absence of a priori statistical information about the input and measurement noise. As a direct modification of the KF, the AKF is distinguished by sufficiently high accuracy and at the same time the simplicity of implementation in a digital computer.

## 6. Adaptive Estimation Algorithm under Conditions of Abnormal Measurements

However, when abnormal measurements appear in the GNSS signals, the conventional adaptive Kalman filters mentioned in Section 5 do not allow for the effective correction of the UV SINS. GNSS signals are exposed to active and passive interference. On average, forty percent of GNSS measurements are abnormal. Under favorable weather conditions, in the absence of active interference, ten percent of GNSS measurements will appear as abnormal measurement points [27,44]. Penetrating the estimation of SINS errors, abnormal measurements reduce the accuracy of correction and, accordingly, the SINS accuracy of determining navigation information.

When restoring the GNSS signal after losing connections, the appearance of signals is often accompanied by the occurrence of abnormal measurement points. Therefore, when using the correction scheme shown in Figure 2, the errors increase sharply in the estimation algorithm. The decrease in estimation accuracy is due to the abnormal measurements. When abnormal measurements significantly exceed the level of measurement of the information sample, the estimation becomes unreliable.

In this section, we propose an adaptive estimation algorithm equipped with a relay selection of the formula for calculating the estimate of $x_{k}$. When an abnormal measurement occurs, the maximum theoretically predicted value will be used instead of the innovation sequence (Formula (9)).

In order to quickly and efficiently detect the abnormal measurements, a criterion based on the innovation sequence is introduced here [24]:

(8) $$tr\left(v_{k+1}v_{k+1}^{T}\right)\leq \gamma \cdot tr\left[H_{k+1}P_{k+1|k}H_{k+1}^{T}+R_{k+1}\right]$$

where γ is the level coefficient of the abnormal measurements, tr is trace operator denoting the sum of the diagonal elements of the matrix, *v* is the innovation sequence, $H_{k+1}$ is the observation matrix, $P_{k+1|k}$ is the a priori covariance matrix of estimation errors, and $R_{k+1}$ is the covariance matrix of measurement noise.

The selection of the level coefficient of the abnormal measurements is based on the following considerations: The random value of the innovation process should not exceed its triple RMS value. Typically, the coefficient is selected from the range γ=7–9. In practical applications, measurement levels that are considered to be abnormal can be adjusted by changing the coefficient γ.

In order to reduce the influence of abnormal measurements on the estimation, we propose a method of replacing innovation $v_{k}$ with limited values ${\left[\gamma \left(H_{k+1}P_{k+1|k}H_{k+1}^{T}+R_{k+1}\right)\right]}^{1/2}$ when abnormal measurements are detected. Then, the estimate of the state vector will have the following form:

(9) $${\hat{x}}_{k+1}=\Phi_{k+1,k}{\hat{x}}_{k}+K_{k+1}{\left[\gamma \left(H_{k+1}P_{k+1|k}H_{k+1}^{T}+R_{k+1}\right)\right]}^{1/2}$$

In this formula, the estimation accuracy is increased not by excluding abnormal measurements from the estimation process, but by limiting the abnormal measurements.

Combined with the adaptive Kalman filter mentioned in Section 3, the new adaptive estimation algorithm that can work under the conditions of abnormal measurements is proposed as follows:

(10) $$\begin{array}{c} v_{k+1}=z_{k+1}-H_{k+1}\Phi_{k+1,k}{\hat{x}}_{k}\\ P_{k+1|k}=\Phi_{k+1,k}P_{k}\Phi_{k+1,k}^{T}+K_{k}E[v_{k}v_{k}^{T}]K_{k}^{T}\\ K_{k+1}=\left\{\begin{array}{c} P_{k+1|k}H_{k+1}^{T}{\left[H_{k+1}P_{k+1|k}H_{k+1}^{T}+R_{k+1}\right]}^{-1}\\ P_{k+1|k}H_{k+1}^{T}{\left[E\left(\hat{v_{k+1}v_{k+1}^{T}}\right)\right]}^{-1} \end{array}\right.\mathrm{when}\left\{\begin{array}{c} diag\left[E\left(\hat{v_{k+1}v_{k+1}^{T}}\right)\right]\leq diag\left[H_{k+1}P_{k+1|k}H_{k+1}^{T}+R_{k+1}\right]\\ diag\left[E\left(\hat{v_{k+1}v_{k+1}^{T}}\right)\right]>diag\left[H_{k+1}P_{k+1|k}H_{k+1}^{T}+R_{k+1}\right] \end{array}\right.\\ {\hat{x}}_{k+1}=\left\{\begin{array}{c} \Phi_{k+1,k}{\hat{x}}_{k}+K_{k+1}v_{k+1}\\ \Phi_{k+1,k}{\hat{x}}_{k}+K_{k+1}{\left[\gamma \left(H_{k+1}P_{k+1|k}H_{k+1}^{T}+R\right)\right]}^{1/2} \end{array}\right.\mathrm{when}\left\{\begin{array}{c} tr\left(v_{k+1}v_{k+1}^{T}\right)\leq \gamma \cdot tr\left[H_{k+1}P_{k+1|k}H_{k+1}^{T}+R_{k+1}\right]\\ tr\left(v_{k+1}v_{k+1}^{T}\right)>\gamma \cdot tr\left[H_{k+1}P_{k+1|k}H_{k+1}^{T}+R_{k+1}\right] \end{array}\right.\\ P_{k+1}=(I-K_{k+1}H_{k+1})P_{k+1|k} \end{array}$$

where *I* is the identity matrix and $\Phi_{k+1,k}$ is the matrix of the SINS error model [28,43,45].

The original feature of this algorithm is the relay selection of the formula for estimating *x*. If an abnormal measurement is detected, the estimate is calculated using the theoretically predicted value of the innovation sequence. If the criterion does not detect an abnormal measurement, then the estimation is calculated as in the conventional adaptive algorithm (Equation (6)). The new adaptive Kalman filter can operate in the absence of accurate prior noise statistics and in abnormal measurement conditions.

## 7. Simulation Results

**Figure 3:**
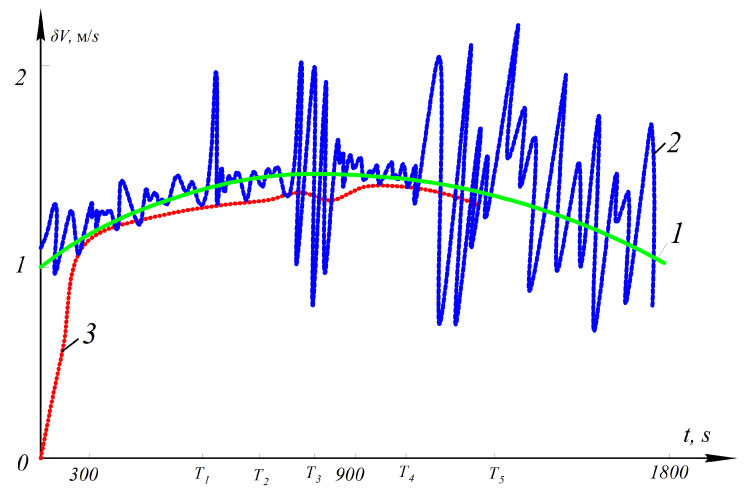
SINS errors in determining the speed and its estimation obtained by the proposed AKF: 1—SINS error in determining the speed; 2—measurements z, which are a mixture of SINS and GNSS errors; 3—SINS error estimation using the proposed AKF.

In order to test the effectiveness of the proposed AKF, a mathematical simulation of SINS error estimation was carried out. The SINS error model is Equation (1). The estimation of SINS errors in determining the speed using the proposed AKF is presented in Figure 3.

At time T1, an abnormal outlier appears in the measurements. In this case, the estimate remains practically unchanged. This is because the abnormal measurement is detected and suppressed and does not affect the estimate. A method to determine the occurrence of abnormal measurements is matching the variance of the innovation sequence to its theoretically predicted value [28,46]. In the time interval T2–T3, a series of abnormal measurements occur, and the estimation accuracy decreases. This is due to the fact that when abnormal measurements occur, the estimates cannot be properly corrected by newly incoming measurements. From the moment of time T4, the measurement noise level increases; all measurements become abnormal. Initially, in the absence of effective measurement information, the estimation accuracy decreases. Then, from time T5, the estimation algorithm cannot work without normal measurements. In this case, various methods can be used to forecast the estimates of the state vector, but the accuracy will be lower than the SINS/GNSS integrated navigation system [28].

## 8. Experimental Tests

In order to verify the effectiveness of the proposed algorithm, several tests were carried out on urban roads in Moscow. A small-sized unmanned vehicle developed by the Department of Automatic Control Systems of Bauman Moscow State Technical University was used as the test vehicle in this experiment. The test vehicle traveled along a fixed route around the university. The coordinates of the route were known in advance from the map. The length of the route was 2 km. The vehicle moved at a speed of 30 km/h. The experiment used the MEMS-SINS GL VG 109, and a built-in GNSS receiver was included in the SINS device.

**Figure 4:**
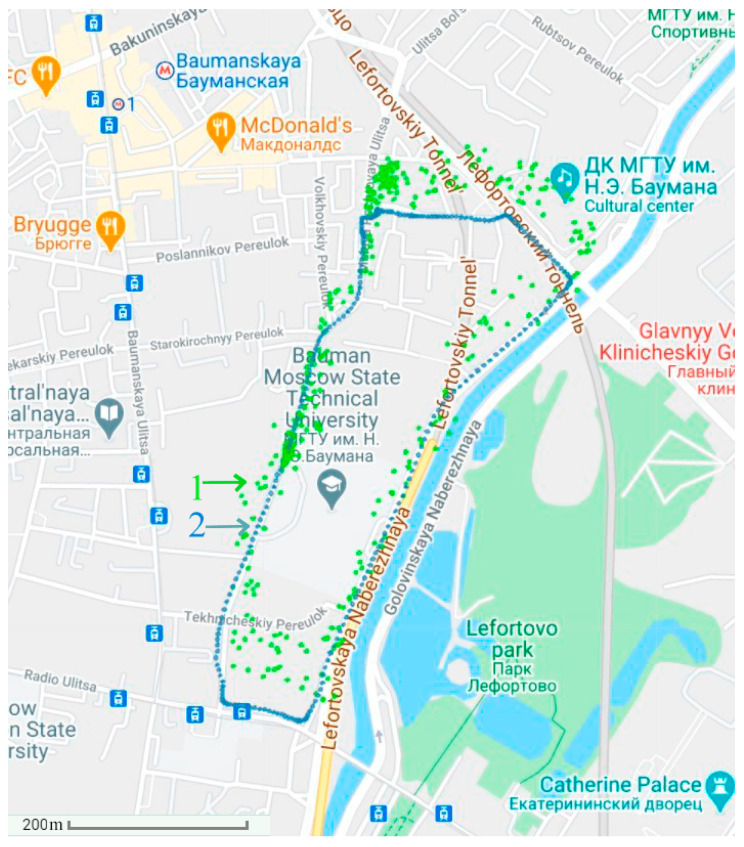
The positioning results of autonomous SINS: 1—positioning results; 2—actual path.

**Figure 5:**
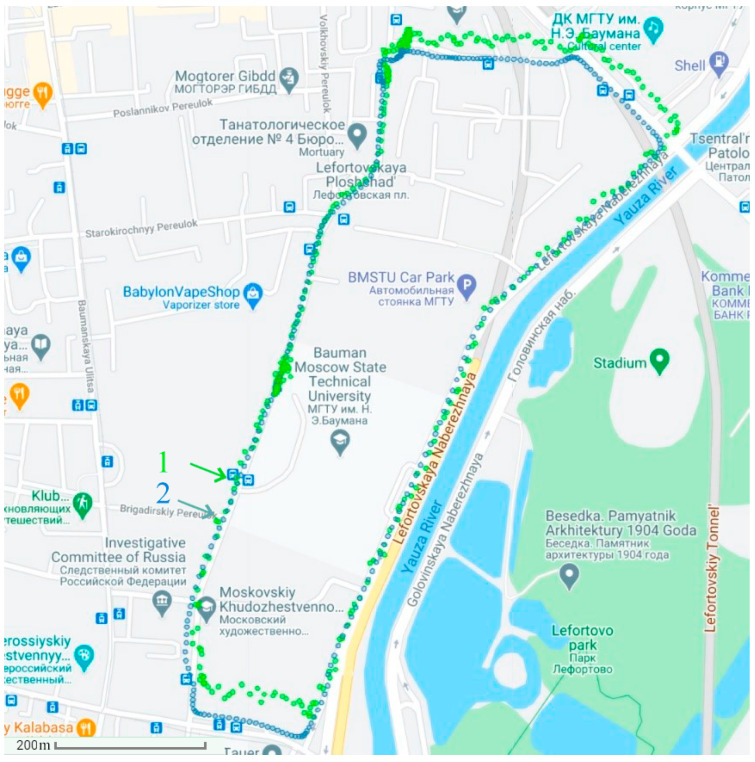
The positioning results of the SINS corrected by the GNSS (containing 15% abnormal measurements) using the KF: 1—positioning results; 2—actual path.

**Figure 6:**
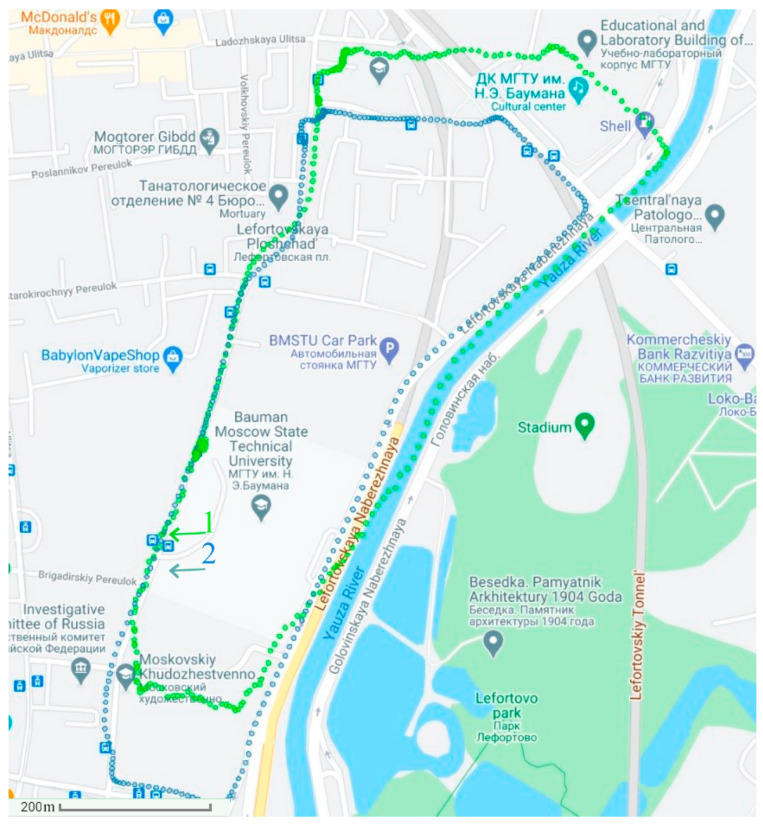
The positioning results of the SINS corrected by the GNSS (containing 40% abnormal measurements) using the KF: 1—positioning results; 2—actual path.

**Figure 7:**
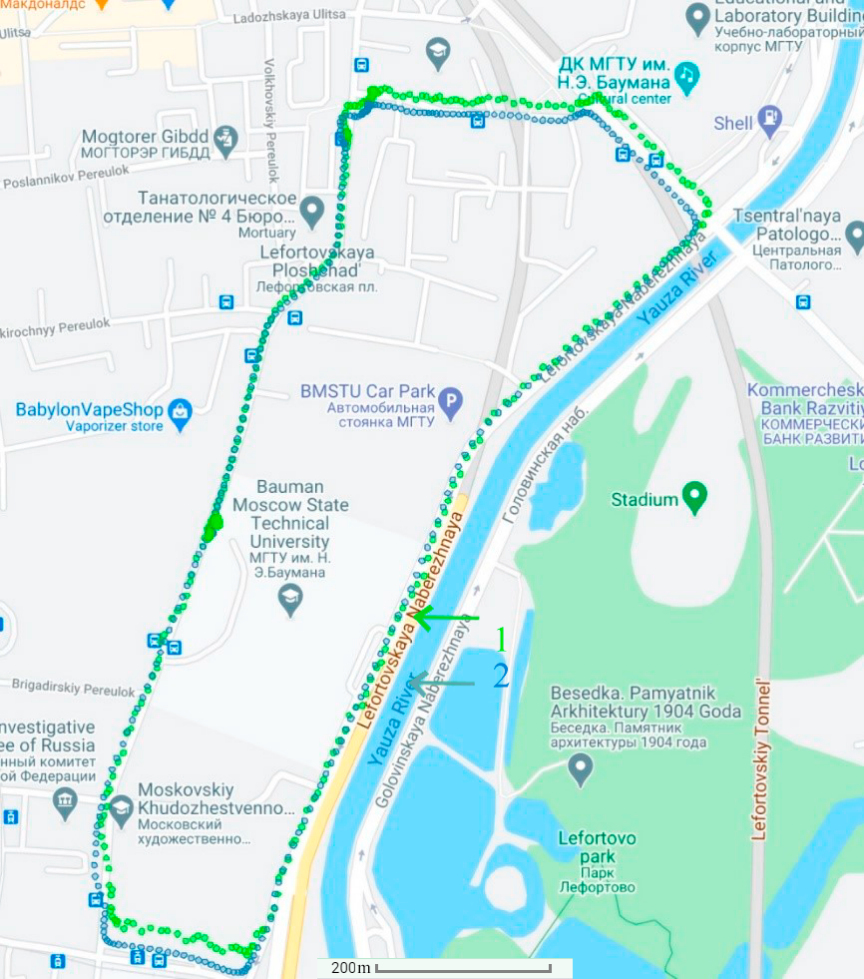
The positioning results of the SINS corrected by the GNSS (containing 15% abnormal measurements) using the proposed AKF:1—positioning results; 2—actual path.

The experiments were carried out at 9:00 a.m. under the conditions of 15% abnormal measurements and at 11:00 a.m. under the conditions of 40% abnormal measurements (due to active interference). First, in Figure 4, the determination of the vehicle’s position uses the autonomous SINS without the GNSS. Then, in Figure 5 and Figure 6, the position of the vehicle is determined by the integrated SINS/GNSS with 15% and 40% abnormal measurements, respectively. The information of the SINS is corrected by the GNSS signal using the conventional Kalman filter. Finally, in Figure 7, the vehicle’s position is determined by the SINS and GNSS with 15% abnormal measurements using the proposed adaptive estimation algorithm.

The experiment was divided into 4 groups, and a total of 100 experiments were conducted. For each group, twenty-five experiments were performed under the corresponding experimental environment, equipment, and algorithm conditions, and the results were obtained from the average of the 25 experiments. The experimental results are shown in Table 4.

As can be seen, the positioning performance of the autonomous SINS was the worst. The positions corrected by the GNSS signal using the KF had higher accuracy. The positioning result corrected by the GNSS signal with 40% abnormal measurements was worse than 15% abnormal measurements, but it was still slightly better than the autonomous SINS. The correction scheme using the proposed AKF and the criterion for evaluating the abnormal measurement levels could significantly eliminate the influence of the abnormal measurements and had the best estimation accuracy. This was due to the fact that in the conditions of an increased level of abnormal measurements, the maximum theoretically predicted value of the innovation sequence was used instead of the actual innovation sequence value when calculating the estimates in the proposed AKF.

In addition, in order to verify the effectiveness of the modification of the AKF, a comparison between the conventional AKF (Formulas (5)–(7)) and the proposed AKF (Formula (10)) was made. The average accuracy of the estimation algorithms is presented in Table 5.

## 9. Conclusions

A study of the small-sized low-accuracy SINS installed on unmanned vehicles with limited life cycles is conducted in this paper. A new adaptive SINS correction algorithm that can work in the absence of accurate prior statistical information and under the conditions of abnormal measurements was developed. The algorithm uses the criterion for identifying abnormal measurements to assess the quality of the information and prevent the external environmental interference from affecting the estimation algorithm, which is very important for unmanned vehicles. Thus, instead of using the SINS of the third accuracy class, it became possible to use a much less expensive SINS with algorithmic correction and achieve the same accuracy. The simulation and experiment results show that with the help of the GNSS and the proposed estimation algorithms, navigation information could achieve a higher accuracy class with a low-cost low-accuracy SINS. In future work, we will consider the identification of the parameters and structure of the SINS error model during intensive maneuvering. 

## Figures and Tables

**Table 1:** Positioning accuracy of different estimation algorithms.

Estimation Algorithms	RMS, m
without filter	20
median filter	11.7
Tukey 53X procedure	9.4
moving average algorithm	6.5
adaptive Kalman filter	4.2

**Table 2:** The parameters of the MEMS-SINS GL VG 109. ADC, is air data computer.

Channel (σ), ${}^{\circ }$	Characteristic Parameters
yaw angle	50${}^{\circ }$
roll, pitch	0.4${}^{\circ }$ (with odometer/ADC/GNSS); 1.0${}^{\circ }$ (without odometer/ADC/GNSS)
accuracy of dead reckoning (odometer/ADC/GNSS)	5% (of the traveled distance within 60 s after losing data from the GNSS)
yaw angle increment error	0.8${}^{\circ }$

**Table 3:** Comparison of the properties of the SINS and the GNSS.

System	Advantages	Disadvantages
SINS	autonomy; high output rate of coordinates, speed, and orientation angles; the possibility of developing dynamic parameters; hardware duplication	accumulation of errors; the problem of initial calibration; the problem of determining the course at the pole; dependence of the accuracy on gravitational anomalies
GNSS	no accumulative errors; no dependence on gravitational anomalies; short readiness time; no dependence on latitude; time measurement	low speed issuing navigation parameters; low noise immunity; signal loss (shading); integrity issue; initial ambiguity of phase measurements; loss of an integer number of periods

**Table 4:** Positioning errors of different methods.

Navigation Systems	Positioning Errors, m
autonomous SINS	3.16
SINS+GNSS (15% abnormal measurements) using KF	1.53
SINS+GNSS (40% abnormal measurements) using KF	2.84
SINS+GNSS (15% abnormal measurements) using proposed AKF	0.95

**Table 5:** The positioning accuracy of different estimation algorithms.

Estimation Algorithms	RMS, m
Conventional AKF (15% abnormal measurements)	1.15
Proposed AKF (15% abnormal measurements)	0.95
Conventional AKF (40% abnormal measurements)	2.2
Proposed AKF (4% abnormal measurements)	1.4

## Data Availability

Data sharing not applicable.
